# Relationship between the roots of *Hippophae rhamnoides* at different stump heights and the root microenvironment in feldspathic sandstone areas

**DOI:** 10.7717/peerj.14819

**Published:** 2023-01-27

**Authors:** Lu Liu, Yuefeng Guo, Xiaoyu Liu, Yunfeng Yao, Wei Qi

**Affiliations:** 1 Inner Mongolia Agricultural University, Hohhot, China; 2 Inner Mongolia Autonomous Region Water Conservancy Development Center, Hohhot, China

**Keywords:** Feldspathic sandstone areas, Vegetation restoration, *Hippophae rhamnoides* root, Root microenvironment, Stump height

## Abstract

**Background:**

To solve the withering of *Hippophae rhamnoides* plantation in the feldspathic sandstone areas of Inner Mongolia and to promote the regeneration, rejuvenation, and sustainability of *H. rhamnoides* forests.

**Methods:**

We stumped aging *H. rhamnoides* trees at the ground heights of 0, 10, 15, and 20 cm (S1, S2, S3, and S4, respectively) and utilized unstumped trees as the control (CK). We then analyzed the effects of the different stump heights on the roots and the root microenvironment of *H. rhamnoides* and the relationship between the roots and the root microenvironment in the stumped *H. rhamnoides*.

**Results:**

The root fractal features, root branching rate, root length, root soluble proteins, soil moisture content, and soil nutrients among the different treatments were ranked as S3>S2>S1>S4>CK (*P* < 0.05). The root topological index, root proline, and malondialdehyde among the different treatments were ranked as S3<S2<S1<S4<CK (*P* < 0.05). The topological indices of S1, S2, S3, S4, and CK were 0.80, 0.86, 0.89, 0.94, and 0.98, respectively, and all were near 1. This result indicated a typical fishtail-shaped branching structure. The root length and root fractal dimensions were primarily affected by the positive correlation of the soil moisture content and the soil organic matter, and the root topological index was primarily affected by the negative correlation of the root proline. Root nutrients were dominant in the changes in the root architecture, while soil moisture and nutrients played supporting roles. These results indicated that stumping can promote plant root growth and root nutrient accumulation, thereby improving soil moisture and the soil nutrient distribution, and the S3 treatment had the greatest impact on the *H. rhamnoides* roots and root microenvironment. Therefore, the 15 cm stump height treatment should be implemented for withering *H. rhamnoides* in feldspathic sandstone areas to promote vegetation restoration.

## Introduction

Roots are the main organs of plants that absorb moisture and nutrients and play key roles in the exchange of matter and energy between plants and soils (Gebre & Earl, 2021; Ong et al., 2002). The architecture properties of its roots reflects the spatial distribution characteristics of roots in a growth medium. These properties can be described according to the geometrical morphological parameters and topological indices of the roots (Pervaiz et al., 2020). These architecture properties directly affect the nutrient and water absorption migration efficiency of *H*. *rhamnoides* from the soil, thereby affecting the growing conditions of the aboveground portion of *H*. *rhamnoides*. Because the underground growth of roots cannot be visually understood, and complete roots cannot be easily acquired, researchers have used the shape, root crown ratio, and root weight to reflect root growth, but only rarely have studied the branching or structural characteristics of the roots (Chun et al., 2021; Liu et al., 2022).

The feldspathic sandstone zones in Inner Mongolia have the most severe soil erosion in the Loess Plateau of China (and in the world). Feldspathic sandstone has a low structural strength that is, it is as hard as rock in the absence of water and is as soft as mud upon contact with water. However, it becomes loose and corrosive upon contact with wind (Wang, Yan & Wang, 2020; Yang et al., 2013), which along with other adverse environmental factors (*e.g*., local dry climate and low annual rainfall), make it extremely prone to erosion. *Hippophae rhamnoides* (Elaeanaeeae, *Hippophae*) is a critical soil-water conservation plant found in arid and semiarid zones. With drought, this shrub can propagate quickly because of its flourishing roots and strong tillering and germinating ability (Zhang, Xu & Wang, 2009). It has excellent soil-water conservation abilities and is predominant in the feldspathic sandstone areas of Inner Mongolia (Yang et al., 2014). Along with the steady progression of key statewide administration projects in feldspathic sandstone areas, the cultivation of *H*. *rhamnoides* has continually improved the ecological and economic benefits of feldspathic sandstone areas. *H*. *rhamnoides* plantations, however, will largely wither in both growth and productivity as they near the age of 10 years (Guo et al., 2020; Tang et al., 2013). Therefore, to reduce the decreasing rate of *H*. *rhamnoides* in feldspathic sand areas and promote the growth of *H*. *rhamnoides* as well as improve the soil properties under the forest, restoration activities in *H*. *rhamnoides* forests (*e.g*., stumping) to improve vegetation and ecological restoration in the feldspathic sandstone areas are of great importance (Liu et al., 2022).

Stumping is an important measure to stimulate branch germination during which seedlings are cut flat at above ground or above a certain height. Stumping has a low cost and high efficiency, and it contributes to the efficient use of soil moisture by plants, Furthermore, it promotes the regeneration, rejuvenation, and sustainability of plants (Dinh et al., 2019; Hamilton et al., 2013). As a result of the destruction of the aboveground tissue of the plant after stumping, the root–crown ratio becomes unbalanced and the plants will grow to compensate for this loss (Fang et al., 2006; Giambalvo, Amato & Stringi, 2011; Marden, Lambie & Rowan, 2018; Wang et al., 2014), using the nutrients stored in its roots to support the growth of the aboveground tissue. Therefore, the germinating branches grow rapidly and increase the plant’s regeneration ability (Bandyopadhyay et al., 2016). Studies have shown that the ability of *H*. *rhamnoides* to preserve water and soil primarily depends on the ability of the roots to enhance soil infiltration and antierosion properties (Zhang et al., 2020). The ability of *H*. *rhamnoides* to preserve water and soil in feldspathic sandstone areas is closely related to the architecture properties of its roots in the soil.

The relevant research to data, however, has been somewhat limited to coppicing or sprouting, in particular, the relationship of the sprouting branch number with the biomass increment and stump height (Langworthy et al., 2019; Yang et al., 2020). Research on the influence of the stump height on the sprouting ability of roots, as well as the relevant mechanism, is lacking. In addition, the growth and development of roots is a self-regulation mechanism, and the self-regulation of root architecture is closely correlated to environmental factors. Hence, research on roots and the root microenvironment is very important (Kaarakka et al., 2018).

To address this research gap, We studied root architecture, nutrients, and the microenvironment of *H*. *rhamnoides* at different stump heights. We hypothesized that roots and the root microenvironment of *H*. *rhamnoides* after stumping were all significantly better than the unstumped trees, and stumping improved the original branch structure and microenvironment of roots, to promote plant growth. These findings contribute to a more comprehensive understanding of the overall functions and environmental adaptability of *H*. *rhamnoides* in feldspathic sandstone areas. The findings also provide a theoretical basis for revegetation, efficient soil-water-fertilizer use, and soil-water loss prevention and control in feldspathic sandstone areas.

## Materials and Methods

### The study area

The study area (39°42′–39°50′N, 110°25′–110°48′E) was located in the soil-water conservation science demonstration plot in Nuanshui Village, Jungar Banner, Ordos, Inner Mongolia. This area has a complex topography, ravines and folds, and undulating beam bases, so it is easy to suffer soil erosion and water loss. It has an average altitude of up to 800–1,590 m, with precipitation of 400 mm, concentrated in July and August, and the average annual temperature is 6.2–8.7 °C. The duration of sunlight is more than 300 d, and the average evaporation is 2,093 mm annually. Artificial vegetation in this area is mainly H. *rhamnoides*, *Pinus tabuliormis*, *Caragana korshinskii*, *Medicago sativa*, and *Prunus sibirica* (Liu et al., 2022).

### Experimental design

In the demonstration plot, we selected a wintering *H*. *rhamnoides* plantation that had fairly consistent site conditions and forest compositions as the experimental sites. These were under a northwest slope face of 4°. On the same sloping face, trees were planted at a row spacing of 2 m × 4 m. The trees were stumped in early March, 2020. The stump heights were 0, 10, 15, and 20 cm above the ground (treatments S1, S2, S3, and S4, respectively). A plantation site without stumping was established as the control (CK). All of the sites were 50 m × 50 m in area, and each treatment was conducted in triplicate. In each site, we selected the standard trees according to the statistics of the tallied results. Stumping was conducted using electric saws and pruning shears, and this ensured that the incisions were flat and smooth without burrs. The stumping mode was complete stumping. To decrease the moisture dissipation, we conducted painting after the stumping. In the middle of August 2021, we surveyed the standard trees in each site for the height and crown widths (Table 1) and then selected five typical root clusters under fairly consistent conditions for the root and soil measurements. A total of 75 root clusters were chosen.

**Table 1:** Information of the sample sites.

Stump height	Forest age (years)	Average tree height (cm)	East–west crown diameter (cm)	South–north crown diameter (cm)
S1	10	72.01 ± 1.87d	53.18 ± 2.52c	52.59 ± 1.32c
S2	10	85.26 ± 2.16b	62.79 ± 2.94b	60.71 ± 1.62b
S3	10	87.12 ± 1.96b	63.32 ± 1.58b	61.01 ± 2.04b
S4	10	79.32 ± 2.07c	59.98 ± 2.23b	57.37 ± 1.93b
CK	10	108.91 ± 2.13a	92.31 ± 2.38a	84.23 ± 1.87a

**Note:**

Values are means ± SD. Significant differences are indicated by different lowercase letters at *P* < 0.05.

### Root architecture properties

We collected the roots using the root tracing method and the whole root digging method. The branches and leaves of samples at different stump heights were cut down from the base, and the weeds and litter around the base were cleaned off. After the surface soils were mostly cleaned, the soils around the roots were carefully cleared away to expose the main roots. We dug the soil along the growing direction of the main roots until reaching the root branches. Digging was continued along the branch roots until reaching the root end. During the sampling, the loss of end high-grade roots was avoided as much as possible to ensure the completeness of the roots (Gao et al., 2020). During the root digging, we marked roots at different soil layer positions at different horizontal distances (0–20, 20–40, 40–60, 60–80, >80 cm) and vertical distances (the soil depths of 0–10, 10–20, 20–30, 30–40, and >40 cm) from the base center of each tree. In addition, we recorded the topological length of the root and the number of different root-level roots to calculate the root topological index and the root branching rate. After the digging, the roots from each stumping treatment were placed flat on the same plane and photographed at the same scale using a high-pixel camera to analyze the root fractal properties. We placed the roots as collected were placed into labeled preservation bags that were put into a refrigerator at 2–3 °C on the same day and then were taken back to our laboratory for the root architecture and nutrient index determinations. Then we returned the soil that was attached to the roots to the laboratory where they were cleaned with deionized water. The roots at different stump heights and different layers and diameters (fine roots <2 mm, coarse roots 2–10 mm, bone roots >10 mm) were scanned using an EpsonScan scanner. We analyzed the root length (RL) and the number of external root connections using the WinRHIZO root analytical system.

#### Fractal properties of the roots

Squared meshes were generated for the root photos at different stump heights using PhotoShop, such that the squared meshes on the side length of the *r*_*i*_ covered the roots (*r*_*i*_ is the side length of the squared mesh for the *i*-th test, *i* = 1, 2, 3…). When the side length of the small square from the *i*-th test was *r*_*i*_ (*r*_*i*_ > *r*_*i*+1_), the number of squares needed to cover the roots was *N*_*i*_. In this way, we generated a group of *r*_*i*_ and the corresponding *N*_*i*_. Then we utilized $\ln {r_i}$ and $\ln {N_i}$ as the x-axis and y-axis, respectively, and obtained the following regression linear equation:

(1) $$\ln {N_i} = - FD\ln {r_i} + \ln K,$$

where *N*_*i*_ is the number of small squares in the side length of *r*_*i*_ needed to cover all of the roots during the *i*-th test; *r*_*i*_ is the scale of the meshing of the root architecture image, namely the side length of the squares in the *i*-th test; *FD* is the fractal dimension of the roots; and $\ln K$ is the root abundance.

#### Topologic index

We calculated this index using the Fitter’s method (Fitter, 1986; Liu et al., 2022):

(2) $$TI = \lg A/\lg M,$$

where *M* is the total number of external connections of the roots; and *A* is the total number of internal connections of the longest root channel. At *TI* = 1, the roots are fishtail-like branches, and at *TI*, which is closer to 0.5, the roots are fork-like branches.

Oppelt proposed the following modified topological index (Liu et al., 2022; Oppelt, Kurth & Godold, 2005):

(3) $$qa = \displaystyle{{a - 1 - Ib{v_0}} \over {{v_0} - 1 - Ib{v_0}}};qb = \displaystyle{{b - 1 - Ib{v_0}} \over {{\raise0.7ex\hbox{${\left( {{v_0} + 1} \right)}$} \!\mathord{\left/ {\vphantom {{\left( {{v_0} + 1} \right)} 2}}\right.} \!\lower0.7ex\hbox{$2$}} - {v_0}^{ - 1} - Ib{v_0}}},$$

where *a*(*A*) is the topological length (number of connections from the base to the root end); *b* is the average topological length, *Ibv*_*0*_ = *lnv*_*0*_/*ln*2, *b* = *Pe*/*v*_*0*_; *v*_*0*_ is equivalent to *M* in Eq. (2); and *Pe* is the total number of channel connections from the root base to the root end. The modified topological indices, *qa* and *qb*, vary within 0–1; *qa* = *qb* = 1 for fishtail-like branches; and *qa* = *qb* = 0 for fork-like branches. The transition between these two modes is that *qa* and *qb* both vary between 0–1.

#### Root branching rate

We determined the root grades from the outside-in according to Strahler’s method (Liu et al., 2022; Strahler, 1952). The roots in the outermost layer were assigned grade 1. The two grade-1 roots met and became a grade-2 root. The two grade-2 roots met and became a grade-3 root. When two roots of different grades met, we assigned the grade of the higher-grade root to the new root. The number *Ni* of roots at grade *I* was counted from the outside in. Then we drew a plot with grade *i* as the x-axis and *lgNi* as the y-axis. On this regression equation, the antilogarithm of the straight slope was exactly the total branching rate (Rb) of the roots.

### Physiological properties of the roots

We measured the physiological indices of the roots according to the *Experimental Guidelines in Plant Physiology* by Wu (2008). We measured each index at a specific soil layer or diameter in triplicate. We detected soluble proteins (SP), proline (Pro), and malondialdehyde (MDA) using the Coomassie brilliant blue method, the sulfosalicylic acid method, and the thiobarbituric acid method, respectively.

### Physicochemical properties of the soils

We collected the soil under the *H*. *rhamnoides* plantation from the sample of each stump height at different horizontal distances (0–20, 20–40, 40–60, 60–80, 80–100 cm) and vertical distances (the soil depths of 0–10, 10–20, 20–30, 30–40, and 40–50 cm) using aluminum boxes and plastic bags. We dried the soil samples taken to the laboratory at 105 °C to constant weights and then the samples were cooled. Then, we measured the dry weights and detected the soil moisture content (SMC) were detected to clarify the soil moisture spatial distributions. We detected soil samples collected in plastic bags were used for the soil nutrient index determination. Soil organic matter (SOM) using dichromate titration coupled with the external heating method (Bao, 2000). We detected nitrate nitrogen (AN), available phosphorus (AP), and available potassium (AK) using phenoldisulfonic acid colorimetry, sodium bicarbonate (NaHCO_3_) extraction and the chroma method, and ammonium acetate (NH_4_OAc) immersed extraction-flame photometry, respectively (Bao, 2000).

### Data processing and analysis

We statistically analyzed the differences in the roots and root microenvironments at different stump heights using a single-factor analysis of variance. We tested significance using Fisher’s least significant difference method. The significance level was *P* < 0.05. Data were analyzed on SPSS 26.0. The spatial distributions of the roots at different stump heights were plotted on Suffer 14.0. The heatmaps of the roots and root microenvironments were drawn on RStudio 1.4.2 and tested using principal component analysis (PCA). We also conducted a redundancy analysis of the root architecture with the root physiology and root microenvironment on Canoco 5.0. Spatial distributions of the SMC were plotted on Origin 9.4.

## Results

### Root architecture properties of *H. rhamnoides* at different stump heights

The root architecture properties were significantly different among the stump heights (*P* < 0.05, Table 2). The topological indices (TI, qa, qb) among the different treatments were ranked as S3<S2<S1<S4<CK, and the TIs were all near 1. This result indicated a typical fishtail-shaped branching structure. In contrast, the TIs after the S3, S2, and S1 treatments were all small (0.80, 0.86, and 0.89, respectively), which indicated that the numbers of secondary branches increased after these treatments and the branch structure became complex. The Rb, fractal dimension (FD), and fractal abundance (lnK) among the different treatments were S3>S2>S1>S4>CK. The Rb, FD, and lnK, which were all maximized after treatment S3, were 2.31, 1.41, and 4.02 of CK, respectively. These results suggested the response to stumping was maximized after treatment S3.

**Table 2:** Topological properties of the roots of *H. rhamnoides* at different stump heights.

Control treatment	TI	qa	qb	Rb	FD	lnK
S1	0.89 ± 0.01CDc	0.62 ± 0.01Dc	0.47 ± 0.01Dc	1.87 ± 0.02Bc	1.14 ± 0.03Cc	3.82 ± 0.02Ac
S2	0.86 ± 0.01CDd	0.54 ± 0.01Dd	0.33 ± 0.01Dd	2.06 ± 0.03Bb	1.23 ± 0.01Cb	3.91 ± 0.01Ab
S3	0.80 ± 0.01CDe	0.38 ± 0.02De	0.19 ± 0.01De	2.31 ± 0.02Ba	1.41 ± 0.01Ca	4.02 ± 0.01Aa
S4	0.94 ± 0.01CDb	0.76 ± 0.01Db	0.50 ± 0.01Db	1.64 ± 0.04Bd	1.02 ± 0.01Cd	3.76 ± 0.01Ad
CK	0.98 ± 0.01CDa	0.92 ± 0.01Da	0.72 ± 0.03Da	1.16 ± 0.02Be	0.85 ± 0.04Ce	3.57 ± 0.02Ae

**Note:**

According to the LSD test, the values of the different lowercase letters in the same column are significantly different, and the values of the different uppercase letters in the same row are significantly different (*P* < 0.05).

**Figure 1:**
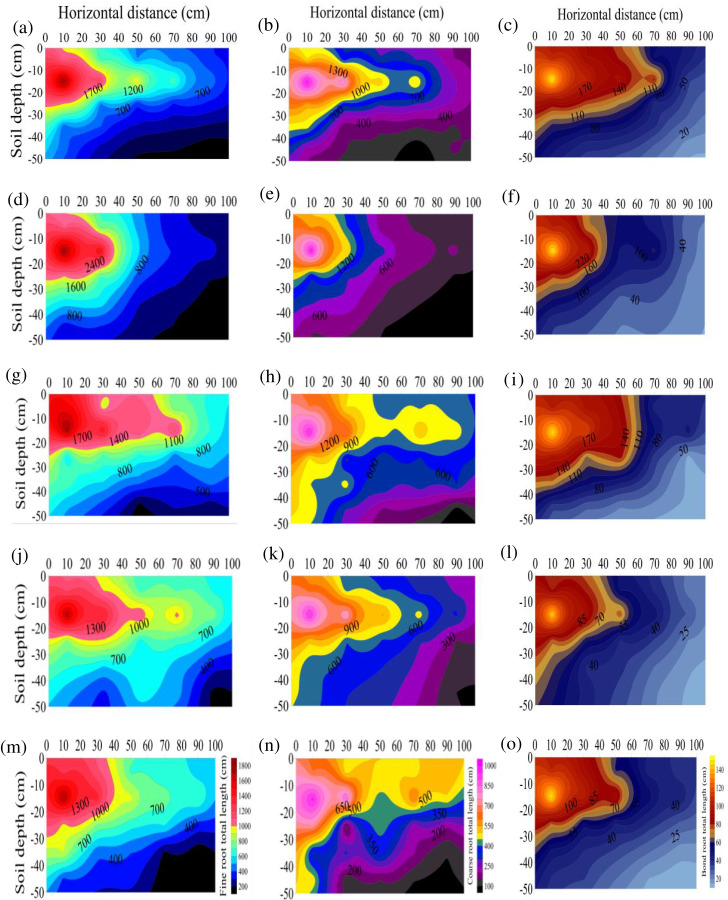
(A–O) Spatial distributions of the total root length at the different root diameters in *H. rhamnoides* at different stump heights.

The RL at the different diameters after any treatment were ranked as S3>S2>S1>S4>CK. The RLs of the S1, S2, S3, and S4 treatments were 1.46, 1.58, 1.66, and 1.27 times that of CK, respectively (Fig. 1), suggesting that stumping could significantly prolong the total root length of *H*. *rhamnoides*, especially after treatment S3. The RL of the different diameters after any treatment ranked as fine roots > coarse roots > bone roots. As the horizontal distance or vertical distance from the root base was lengthened, the total RL at each diameter level in the different treatments rose. The RL were all primarily distributed at a soil depth of 0–30 cm after any treatment and maximized at vertical distance of 10–20 cm and a horizontal distance of 0–20 cm and minimized at a vertical distance of 40–50 cm and a horizontal distance of 80–100 cm.

### Physiological root properties of *H. rhamnoides* at different stump heights

**Figure 2:**
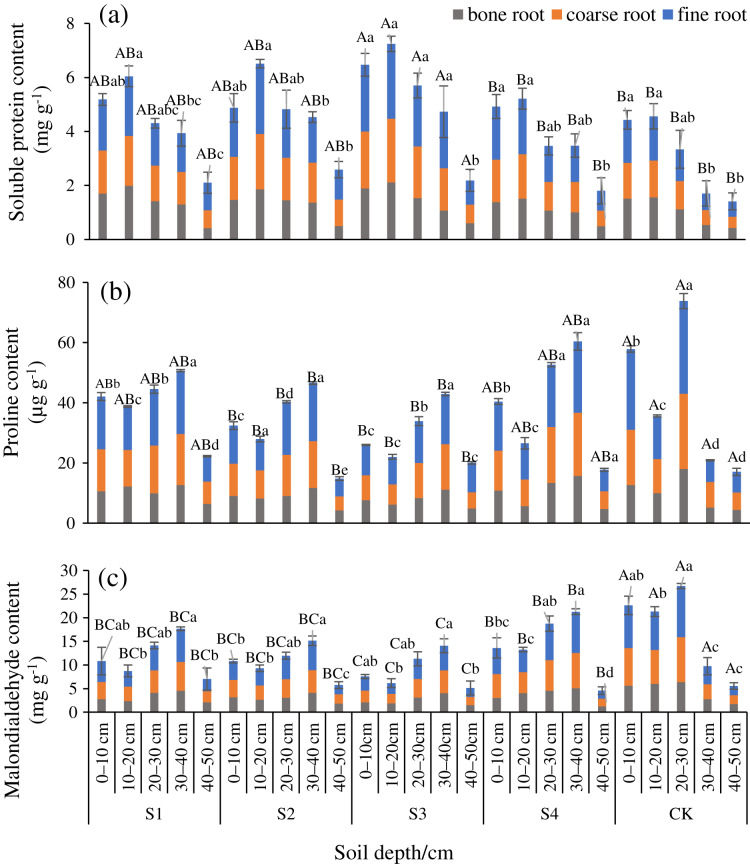
Changes in the root soluble proteins (A), proline (B), and malondialdehyde (C) traits with soil depth of *H. rhamnoides* at different stump heights.

The SP at any diameter level was smaller after any stumping treatment compared with the CK, and Pro and MDA were lower than the CK (Figs. 2A–2C). Differences in the SP, and Pro, MDA between treatments S3 and the CK were significant in the fine roots (*P* < 0.05). The SP of fine roots, coarse roots, and bone roots of treatment S3 *vs* the CK were 1.88, 1.83, and 1.39 times, respectively. These results indicated that all the stumping treatments significantly increased the nutrients in the roots, and the treatment S3 maximally impacted the root nutrients. The SP, Pro, and MDA among the different treatments and the different diameters all ranked as fine roots > coarse roots > bone roots. The SP at all of the diameter levels basically decreased an increase in the soil depth. The distribution characteristics of Pro and MDA were opposite that of SP in each soil layer, and the Pro and MDA at all root diameters first decreased and then increased with each increment in the soil depth.

### Microenvironment properties of *H. rhamnoides* roots at different stump heights

**Figure 3:**
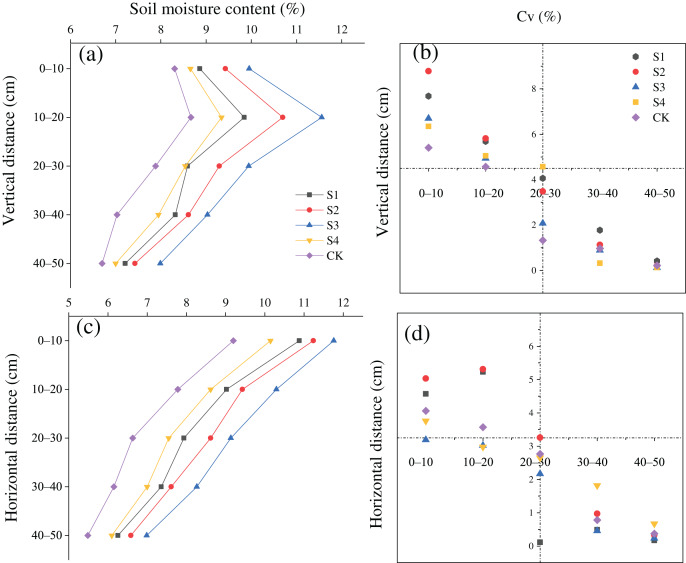
(A–D) Spatial distributions of the soil moisture content of *H. rhamnoides* at different stump heights.

The SMC after treatments S1, S2, S3, and S4 was greater than that in the CK in the vertical and horizontal directions (Figs. 3A and 3C). The SMC after the different treatments first increased and then increased with a rise in the vertical distance from the base, and the moisture content was maximized at a vertical distance of 10–20 cm. In addition, the variation coefficient (Cv) of the SMC was all less than 10% at all soil depths, and the Cv at a depth of 0–20 cm was greater than that at a depth of 20–50 cm (Fig. 3B). The SMC after the different treatments gradually decreased with an increase in horizontal distance. The Cv of the soil moisture content was lower than 10% at all horizontal distances, and the Cv at a depth 0–40 cm was larger than that at a depth of 40–100 cm (Fig. 3D).

**Figure 4:**
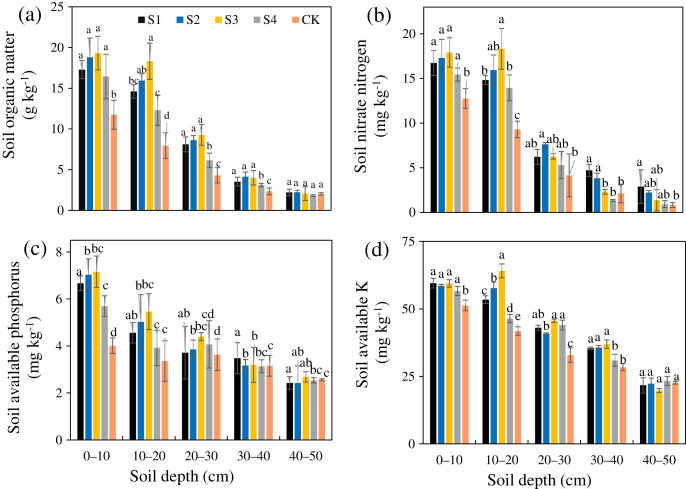
Changes in the soil organic matter (A), nitrate nitrogen (B), available phosphorus (C), and available potassium (D) traits with soil depth of *H. rhamnoides* at different stump heights.

The SOM, AN, AP, and AK after the different treatments were all greater than those in the CK (Figs. 4A–4D). The SOM, AN, AP, and AK of treatment S3 were 1.87, 1.59, 1.37, and 1.28 times that of the CK, respectively, and the increasing amplitudes ranked as SOM>AN>AP>AK. The SOM, AN, AP, and AK after the different treatments all gradually decreased from the surface layer downward. Relative to the CK, the different stumping treatments significantly improved the soil nutrient content at a soil depth of 0–30 cm (*P* < 0.05).

### Relationship between the *H. rhamnoides* roots and the root microenvironment

**Figure 5:**
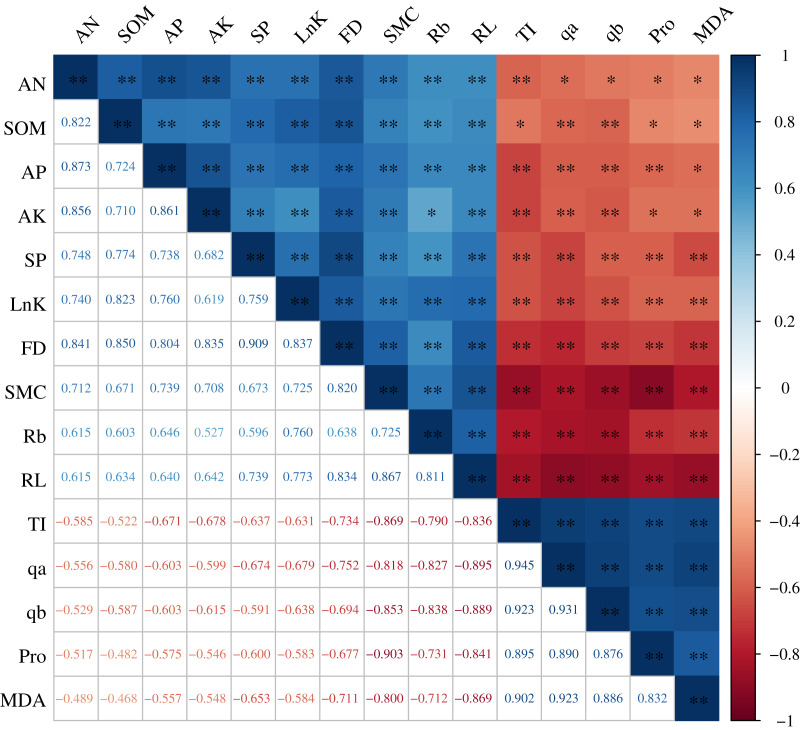
Correlation analysis between the *H. rhamnoides* roots and the root microenvironment.

The root architecture and the root nutrients were significantly correlated with the root microenvironment, and the correlation coefficients (R^2^) were all greater than 0.468 (*P* < 0.01). The root properties (FD, lnK, RL, Rb, and SP) were all significantly and positively correlated with the SOM, AN, AP, AK, and SMC. In particular, the correlation between the RL and SMC and between the FD and SOM were extremely significant, with an R^2^ of 0.867 and 0.850, respectively. TI, qa, qb, Pro, and MDA were all significantly and negatively correlated with the FD, lnK, RL, Rb, SOM, AN, AP, AK, and SMC. The R^2^ between Pro and SMC was maximized at −0.903 and between TI and SMC was maximized at −0.869 (Fig. 5).

**Figure 6:**
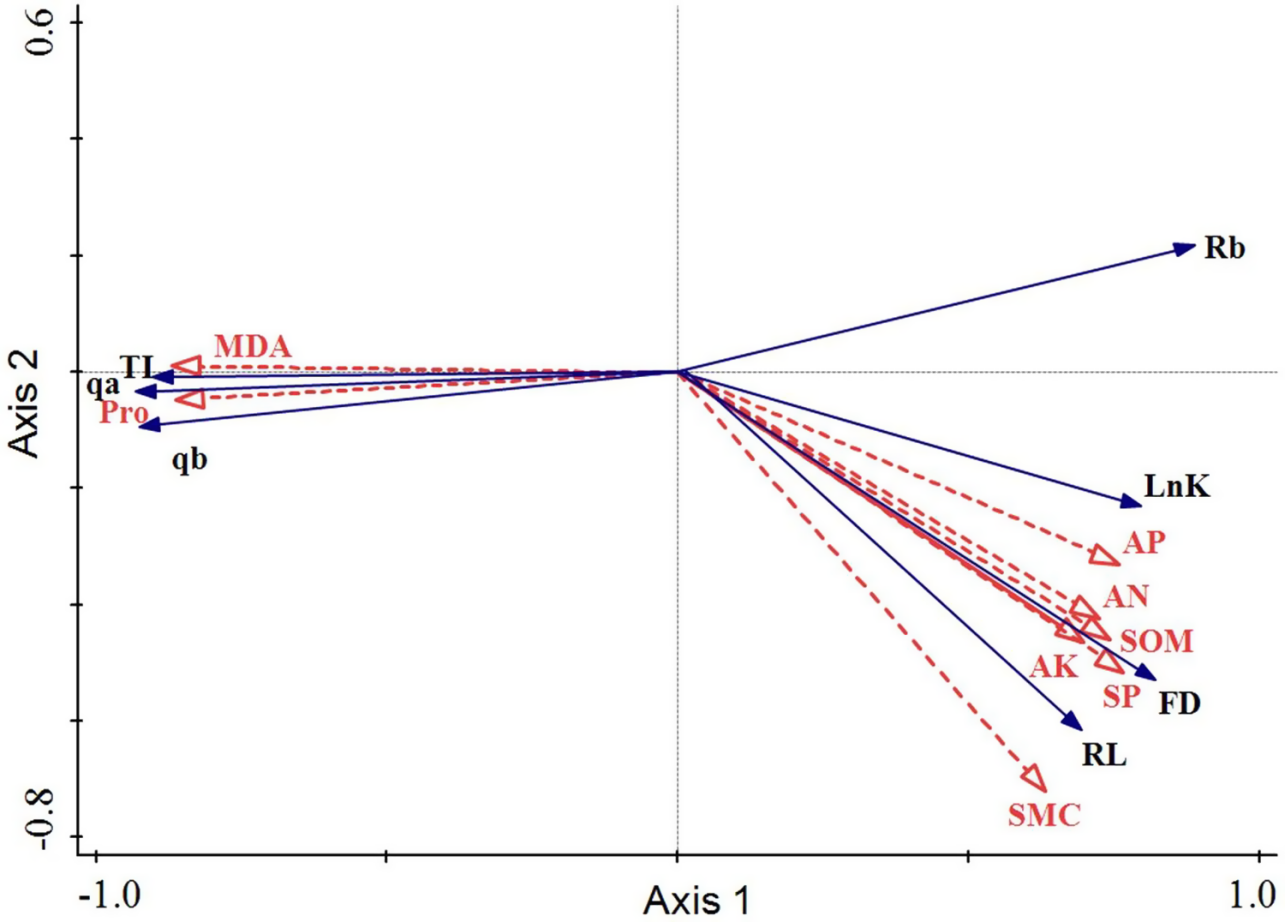
Redundancy analysis of the root architecture of *H. rhamnoides* with the root physiology and microenvironment.

The arrowhead lengths of root physiological and microenvironmental factors reflected the degree of influence of these factors on the response variables. Clearly, the arrowhead lengths of MDA, Pro, SP, and SMC were significantly larger than those of the other explaining variables. Hence, these four factors (MDA, Pro, SP, and SMC) had the most influential effect on changes in the root architecture. The included angles of the arrowheads indicated the correlations of the root architecture with the root physiology and root microenvironment. Thus, AP, AN, SOM, AK, SP, and SMC were all positively correlated with Rb, FD, or RL. MDA and Pro were both negatively correlated with Rb, FD, or RL. These results indicated that the fractal dimension, fractal abundance, and branching rate of the roots all rose as the SMC and soil nutrient content increased. Conversely, as the proline and MDA contents in the roots increased, the roots were most severely threatened by environmental stress and their life was harmed. Hence, the fractal properties of the roots declined, and the space occupying ability of the roots was weakened. The included angles of MDA, Pro, or SP with the first sorting axis were small, which indicated that the root architecture changes were dominant. The included angle between SMC and the second sorting axis was small. The arrowheads of SOM, AP, AN, and AK were all short, and their included angles with the second sorting axis ranked as the angle between SMC and the second sorting axis. Hence, SMC, SOM, AP, AN, and AK all played supporting roles in the changes in the root configuration (Fig. 6).

**Figure 7:**
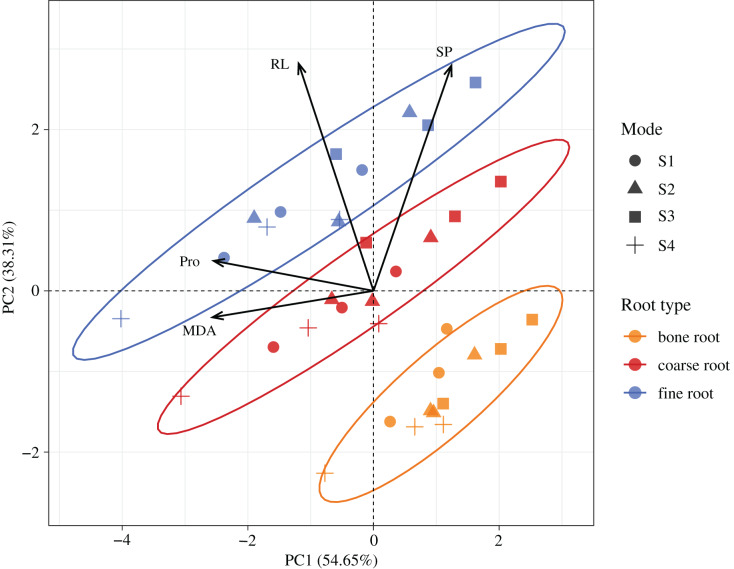
Spatial distributions of the root properties at the different diameters in *H. rhamnoides* at different stump heights.

According to the PCA (Fig. 7), the first two trait axes explained 54.65% and 38.31% of the total variance in the original variables, with an accumulative contribution rate of 92.96%. Hence, we selected the first two trait axes as the active components of the root architecture and root nutrients that explained 92.96% of the information in the original indices and thus were representative. On the first axis, the score of the MDA was the largest, the included angle between the MDA and Pro was the smallest, and the correlation between these two was the highest. On the second axis, the score of the RL was the largest, and its correlation with the SP was the highest. According to the confidence ellipses, the confidence groups were scattered. The trait indices were significantly different among the fine roots, coarse roots, and bone roots. The traits among the different root diameters were distributed from the right lower corner to the upper left corner, and the trend lines had the smallest included angle with the RL followed by Pro. Hence, the major influence factors on the significant differences among the fine roots, coarse roots, and bone roots were the RL and Pro contents.

## Discussion

We applied different stump heights to wintering *H*. *rhamnoides* plantation in the feldspathic sandstone area. We studied the responses of the root topology, root fractal characteristics, root branching rate, and root length to different stump heights. The responses of the growing and physiological properties of the plants to different stump heights are complex processes. Reportedly, plants will grow in a compensatory manner after the aboveground portion is destroyed (Bond & Midgley, 2001). The compensation ability of plants is correlated to the cutting intensity (Hamilton et al., 2013). After stumping, the root biomass increases for certain period of time, as the roots will store abundant starches that can provide nutrition for the aboveground portion to rapidly recover. The roots are an important carrier for material and energy transfer between the soil, plant, and atmosphere interfaces, and the branches and architecture development of roots affect the nutrient absorption and utilization by roots (Wang et al., 2020). Thus, the fractal dimension objectively reflects the structural shapes of the roots and thereby offers a new quantitative indicator to characterize the growth of roots and the entire plant. The fractal dimensions of the roots in regular plants are 1.1–1.9 (Tao, Uma & Hirofumi, 2019). When the fractal dimension is >1.1, the analyzed object can be considered to have obvious fractal features (Wu, 2020).

Our results showed that, except for CK and S4 (root fractal dimension <1.1), the fractal dimensions after all other treatments were greater than 1.1 and were maximized to 1.408 after treatment S3 (Table 2). These results indicated that the root systems of the stumped *H. rhamnoides* in feldspathic sandstone areas were sufficiently fractal. The fractal abundance reflects the expanded root volume in soils and can be used to quantitatively describe the space-occupying ability and moisture and nutrient-absorbing efficiency of the roots (Walk, Van Erp & Lynch, 2004). We found that the root fractal abundance and root lengths of *H. rhamnoides* after stumping was higher than that of the CK (Table 2), which indicated that the root development degree of *H. rhamnoides* was enhanced after stumping. In addition, the number of branches increased, which significantly enhanced the total root lengths and led to a rise in the underground space occupying ability and nutrition competitive ability. All of these factors effectively promoted the growth of both roots and plants.

The root clusters of *H. rhamnoides* after different stumping treatments all formed typical fishtail-shaped branched structures (Table 2), which was consistent with the findings of root systems in six species of slope protecting plants in loess areas (Yang, 2021). Because of certain advantages, the fishtail-shaped branches can effectively avoid internal competition within the roots and thereby rapidly occupy the redundant space (Oppelt, Kurth & Godold, 2005). This redundancy contributes to nutrient absorption and the ability to quickly respond to habitat changes (Oppelt, Kurth & Godold, 2001). The root topological indices after the S3, S2, and S1 treatments were all smaller than after the other treatments, at 0.80, 0.86, and 0.89 respectively (Table 2). This result indicated that the root structure and branch rate became structurally complex after these three stumping treatments and could effectively absorb water and nutrients from the soil. The roots with the different diameters were distributed primarily at a depth of 0–30 cm and especially at 10–20 cm (Fig. 1). After the different treatments, the root lengths of the different diameters decreased with soil deepening. These results were consistent with another study in which the roots of *H. rhamnoides* in bare feldspathic sandstone areas were distributed primarily at a depth of 0–40 cm (Wang, 2018). The roots of *H. rhamnoides* in the feldspathic sandstone areas were distributed primarily in the surface soils that had a shallow root distribution and well-developed horizontal roots. Well-developed horizontal roots effectively occupied the space and efficiently utilized soil moisture and nutrients. Moreover, in feldspathic sandstone areas with frequent wind-blown sand events, rich horizontal roots better fixed and supported the entire plant in comparison with vertical roots (Silva et al., 2010).

Soluble protein concentration is a key indicator of the total metabolism in plants (Chi et al., 2010; Alberton et al., 2013). We found that the SP contents in the different root diameters were all greater after the stumping treatments than with the CK (Fig. 2A). The stumped plants rapidly recovered the aboveground biomass to improve the soil nutrient-absorbing ability of the roots. Moreover, stumping stimulated roots to improve the starch-storing ability and enhanced metabolism to maintain plant growth (Dinh et al., 2019; Xie et al., 2020). Hence, the root SP contents after the stumping treatments were greater than that of the CK. The SP contents after the different treatments and at different diameters gradually decreased with an increase in soil depth. Because soil water and fertilizer levels declined with a decrease in the soil depth, the content of usable resources by roots declined, which led to a decrease in the SP content. All of these levels were maximized at a depth of 10–20 cm after any stumping treatment, which was due to the roots of H. *rhamnoides* living in feldspathic sandstone areas that were distributed in the shallow layers. The roots developed primarily in the 10–20 cm soil layer, which could effectively occupy soil space and absorb soil water and fertilizer (Wang, 2018).

The SP content among the different diameters were ranked as fine roots > coarse roots > bone roots. The SP contents in the fine roots were significantly different among the stumping treatments, but the SP content in the bone roots were slightly different (Fig. 2A). Because the fine roots played a critical role in absorbing nutrients and expanding space (Bardgett, Mommer & De Vries, 2014), they responded significantly to stumping. This then affected the physiological eco-processes related to fine roots. In comparison, the bone roots grew for years and were little affected by 2 years of stumping (Ong et al., 2002). Hence, the SP content in the bone roots was low.

Physiological mechanisms exist for plants to resist unfavorable conditions. Proline, an important organic osmotic regulation substance in cells, plays an important role in balancing the cell osmotic pressure and stabilizing proteins (Kishor et al., 2015). Reportedly, plants under drought will accumulate abundant Pro and other osmotic regulation substances to improve their stress resistance ability (Sofy et al., 2020). MDA, as a product of cell membrane lipid peroxidation, will intensify membrane injury (Johannes & Paul, 2020). Generally, a higher MDA concentration in plants is more harmful to cell membranes.

We found that the Pro and MDA contents at any diameter level were smaller after any stumping treatment compared with the CK (Figs. 2B and 2C). Compared with H. *rhamnoides* after stumping, CK has lush branches and leaves, which require heavy transpiration and large water consumption. To adapt to the dry growing environment, plants produce rich proline to maintain normal osmotic pressure *in vivo*, but excessive proline will introduce certain negative effects on the growth of plants (Kishor et al., 2015; ALHaithloul et al., 2022). We also found that the Pro and MDA at all of the diameter levels among different treatments first decreased and then increased with each increment in the soil depth, and then the Pro and MDA at a depth of 10–20 cm soil layer is on the declined (Figs. 2B and 2C). Because the SMC was high at 10–20 cm, the root system grew well. An enhancement of protective enzymatic activity in the roots moderately inhibited membrane lipid peroxidation (Johannes & Paul, 2020). Thus, Pro and MDA followed a downward trend in this soil layer. At a depth of 20–40 cm, the increasing trend of Pro and MDA was due to the decreasing trend in the soil water content (Figs. 2B and 2C). Because the plants under drought stress generated abundant self-harming active oxygen free radicals, the protective enzymatic activity was inhibited, and the cell membrane lipid peroxidation in the roots was intensified, which led to plasma membrane damage and a large-amplitude increase in the Pro and MDA (Dawood et al., 2022). In addition, the contents of Pro and MDA in the roots of the different treatments and different diameters were lower at 40–50 cm. Because there were very few roots at 40–50 cm, the fine roots were easily damaged and lost activity during sampling and testing. Hence, the values at 40–50 cm were low.

We then analyzed effects of the different stump heights on the root microenvironments of *H*. *rhamnoides* and the relationship between the roots and root microenvironment in the stumped *H*. *rhamnoides*. A decrease in the aboveground leaf area after stumping affected the allocation and transportation of metabolic products and moisture between the aboveground and underground portions, and thereby affected the changes in the SMC (Mooleki et al., 2016; Qi & Guo, 2020). Moreover, the SMC after the different stumping treatments decreased with the lengthening of the horizontal distance from the base (Fig. 3C). First, however, there was an increase and then a decrease along with the rise in the vertical distance from the base, with the maximum depth occurring at 10–20 cm (Fig. 3A). In keeping with these findings, Ma et al. also found that the soil moisture characteristics under typical degraded vegetation in a loess covered feldspathic sandstone area were basically consistent (Ma, 2020). The soil moisture in this region primarily originated from natural precipitation, but the supplemented moisture was basically reserved in shallow soils and barely supplemented the deep soils. Moreover, the majority of precipitation in shallow soils was utilized by plants or evaporated on the ground. This result further explained why the roots were distributed in superficial layers (Yang et al., 2013). Moreover, the 0–10 cm soil layer had more large pores and was affected by surface evaporation, and the water-holding capacity was lower than in the 10–20 cm layer (Wang, 2018). Therefore, the water content was maximized at a vertical distance of 10–20 cm from the standard cluster. Furthermore, the SMC after stumping was greater than that in the CK. Matter was allocated from aboveground to underground after stumping. At this stage, the photosynthesis of *H. rhamnoides* was weakened because of the decrease in the aboveground biomass, and the water consumption and thereby the water volume transportation by roots declined. Hence, the spatial distribution of the soil moisture was significantly improved, but the unstumped *H. rhamnoides* experienced severe transpiration, which caused the roots to absorbed abundant water to support the physiological activities of the aboveground portion. As a result, the soil water content decreased, and the SMC after stumping was greater than that in the CK.

The soil nutrient contents after the different stumping treatments all increased. Specifically, the soils transformed from lean soils to nutritional soils, and the S3 soil nutrients were the largest (Fig. 4). A comparison of the soil nutrients among the different layers showed that the nutrients in the shallow soil were greater than those in the deep soil. The abundant plant litter on the forest land topsoil decomposes to structurally diverse organic matter, which is a major source of SMC (Niu et al., 2021). Additionally, the death metabolism of the roots and the secretion from the roots are also sources of SOM (Wang, Fang & Chen, 2017). Among all of the stumping treatments, the roots grew significantly more at a depth of 10–20 cm, and the root metabolism ability and the root secretions were enhanced, which led to an increase in SOM. Moreover, the nitrogen-fixing root nodules of *H. rhamnoides* were concentrated in the topsoil, and such a distribution improved the nitrogen level in the surface soils of feldspathic sandstone areas and was favorable for soil improvement, soil fertilization, and ground ecological recovery.

Hence, the results of this study showed that stumping promoted plant root growth and root nutrient accumulation, thereby improving the soil moisture and soil nutrient distribution. In addition, the treatment S3 had the greatest impact on the *H. rhamnoides* roots, which holds great significance for the regeneration and rejuvenation of feldspathic sandstone withering *H. rhamnoides*.

## Conclusions

The root architecture of *H. rhamnoides* at different stump heights tended to have a fishtail-shaped branching structure. The root topological index, Pro, and RAD increased significantly after stumping, and Rb, FD, lnK, SP, SMC, and soil nutrients increased. These results indicated that stumping could promote branching and nutrient accumulation of root, thus improving soil moisture and soil nutrient distribution. We also found that stumping had the most significant effect on fine roots and surface soil at a depth of 0–30 cm, and the increasing amplitudes of the soil nutrients ranked as SOM>AN>AP>AK after stumping. This study highlighted the connection between plant roots and the root microenvironment under stump treatment and showed that these effects may not be direct, but rather may cause soil changes through root changes induced by stump treatments. The MDA, Pro, and SP were dominant in the changes of the root architecture, while SMC, SOM, AP, AN, and AK played supporting roles. The RL and Pro were the primary influential factors that caused significant differences among the different root diameters. The root architecture and root microenvironment characteristics at different stump heights were all significantly better than in the unstumped trees, and the optimal stump height was 15 cm. This work plays vital roles in the restoration of vegetation and the prevention of soil erosion in feldspathic sandstone areas.

## Supplemental Information

10.7717/peerj.14819/supp-1 Supplemental Information 1 Sampling site details & root topological properties raw measurements.

## Additional Information and Declarations

### Data Availability

The following information was supplied regarding data availability: The raw measurements are available in the Supplemental File.
